# A1S_2811, a CheA/Y‐like hybrid two‐component regulator from *Acinetobacter baumannii* ATCC17978, is involved in surface motility and biofilm formation in this bacterium

**DOI:** 10.1002/mbo3.510

**Published:** 2017-07-17

**Authors:** Rong Chen, Ruichen Lv, Lisheng Xiao, Min Wang, Zongmin Du, Yafang Tan, Yujun Cui, Yanfeng Yan, Yanping Luo, Ruifu Yang, Yajun Song

**Affiliations:** ^1^ State Key Laboratory of Pathogen and Biosecurity Beijing Institute of Microbiology and Epidemiology Beijing China; ^2^ Department of Clinical Microbiology General Hospital of Chinese People's Liberation Army Beijing China

**Keywords:** *Acinetobacter baumannii *, biofilm formation, motility, pili, quorum sensing, two‐component systems

## Abstract

Two‐component systems in *Acinetobacter baumannii * are associated with its virulence, drug resistance, motility, biofilm formation, and other characteristics. In this study, we used Rec_Ab_, a genetic engineering method, to investigate the function of A1S_2811 in *A. baumannii * strain ATCC17978. A1S_2811, a hypothetical hybrid sensor histidine kinase/response regulator, has four histidine‐containing phosphotransfer domains, a CheA‐like regulatory domain, and a CheY‐like receiver domain at its C terminus. Compared with the ATCC17978 strain, both surface motility and biofilm formation at the gas–liquid interface decreased significantly in the A1S_2811 knock‐out strain. The number of pilus‐like structures and the amount of extrapolymeric substances on the cell surface also decreased in the A1S_2811 null strain. Transcription of *abaI*, which encodes an *N*‐acylhomoserine lactone synthase in *A. baumannii *, decreased significantly in the A1S_2811 null strain, and supplementation with synthetic *N*‐(3‐oxodecanoyl) homoserine‐l‐lactone rescued the surface motility and biofilm formation phenotype in the null mutant. We speculate that A1S_2811 regulates surface motility and biofilm formation, not by regulating type IV pili‐associated genes expression, but by regulating the chaperone/usher pili‐associated *csuA/ABCDE* operon and the AbaI‐dependent quorum‐sensing pathway‐associated A1S_0112‐0119 operon instead.

## 1 INTRODUCTION

*Acinetobacter baumannii *, a ubiquitous, nonfermentative gram‐negative bacterium, is an important infectious, nosocomial pathogen with an extraordinary ability to acquire antibiotic resistance determinants and adapt to hospital environments (Gonzalez‐Villoria & Valverde‐Garduno, 2016). Lots of studies focusing on the dissemination characteristics and mechanisms of antibiotic resistance in *A. baumannii * have been published in the past decades, while investigating the functions of the individual genes and regulatory networks governing its phenotypes has been of great help in gaining better understanding this pathogen.

Bacterial two‐component systems (TCSs) play an important role in regulating the signal transduction of environmental stimuli, including stress conditions (Capra & Laub, 2012). So far, at least five TCSs have been described in *A. baumannii *, including BfmSR (Liou et al., 2014; Tomaras, Flagler, Dorsey, Gaddy, & Actis, 2008), PmrAB (Adams et al., 2009; Beceiro et al., 2011), AdeRS (Marchand, Damier‐Piolle, Courvalin, & Lambert, 2004; Sun et al., 2012), BaeSR (Lin, Lin, Yeh, & Lan, 2014), and GacSA (Cerqueira et al., 2014). These *A. baumannii * TCSs are known to be associated with its virulence, drug resistance, motility, biofilm formation, and other characteristics.

According to the bioinformatics analysis based on the presence of conserved amino acid motifs, structural features or limited homology, A1S_2811 (4,521 bp) in *A. baumannii * ATCC17978 is annotated as a *cheA* homolog (GenBank: NC_009085) (Smith et al., 2007). *CheA* in *Escherichia coli* and its homolog *chpA* in *Pseudomonas aeruginosa*, which are components of the chemotactic signal transduction system in these bacteria, have been investigated in detail (Baker, Wolanin, & Stock, 2006; Elowitz, Surette, Wolf, Stock, & Leibler, 1999; Li, Swanson, Simon, & Weis, 1995; Stewart, 1997; Whitchurch et al., 2004). Both of them are TCSs. It was reported that *cheA/Y* in *E. coli* and *chpA/Y* in *P. aeruginosa* play regulatory roles in controlling bacterial motility via flagella or type IV pili (Alon et al., 1998; Baker et al., 2006; Bertrand, West, & Engel, 2010; Elowitz et al., 1999; Li et al., 1995; Whitchurch et al., 2004); however, A1S_2811 in *A. baumannii * has not been studied as yet.

Although it lacks flagella, *A. baumannii * is motile (Clemmer, Bonomo, & Rather, 2011; Mussi et al., 2010). The underlying molecular and genetic basis of motility in *A*. *baumannii *’ remains ambiguous (McBride, 2010), and its motility phenotypes are diverse. Certain *A. baumannii * strains exhibit a phenomenon known as twitching motility, which is visualized as a kind of jerky movement on wet surfaces (Eijkelkamp et al., 2011b; Semmler, Whitchurch, & Mattick, 1999). Barker & Maxted (1975) observed movements on the wet surface of semisolid media, which they called “swarming” movement; they also noticed that when *A. baumannii * was inoculated by stabbing it into agar, some strains could move beneath the agar or form “ditches.” A series of genes required for the surface motility of *A. baumannii * have been identified (Clemmer et al., 2011), and the motility was associated with type IV pili (Clemmer et al., 2011; Harding et al., 2013), quorum sensing (Clemmer et al., 2011), blue light sensing (Mussi et al., 2010), iron deficiency (Eijkelkamp, Hassan, Paulsen, & Brown, 2011a), and 1,3‐diaminopropane (Skiebe et al., 2012). However, the published literature has insufficiently answered the question of how *A. baumannii * moves, and even the definitions of the various forms of movement are confusing. Therefore, investigating the A1S_2811 hypothetical chemotactic signal transduction system component in *A. baumannii * ATCC17978 has potential to contribute not only to better understanding of the function of TCSs in *A. baumannii * but also to elucidate its motility mechanism.

## 2 MATERIALS AND METHODS

### 2.1 Bacterial strains and plasmids

The bacterial strains and plasmids used in this study are listed in Table 1. Bacterial strains were routinely maintained in Luria–Bertani (LB) broth or agar.

**Table 1:** Bacterial strains and plasmids used in this study

Strain or plasmid	Characteristic (s)	Source or reference
Strains		
*A. baumannii * strain ATCC17978	Reference strain	ATCC
17978‐pAT01	*A. baumannii * 17978 carrying pAT01	Tucker et al.
17978‐pAT02	*A. baumannii * 17978 carrying pAT02	Tucker et al.
17978‐pBAD18Kan‐ori	*A. baumannii * 17978 carrying pBAD18Kan‐ori	This study
17978‐pABBR_MCS	*A. baumannii * 17978 carrying pABBR_MCS	This study
Δ2811::Kan^r^	*A. baumannii * 17978 Δ2811::Kan^r^	This study
Δ*csuE*::Kan^r^	*A. baumannii * 17978 Δ*csuE*::Kan^r^	This study
Δ2811::FRT	*A. baumannii * 17978 Δ2811::FRT	This study
Δ*csuE*::FRT	*A. baumannii * 17978 Δ*csuE*::FRT	This study
Δ2811‐pBAD18Kan‐ori	*A. baumannii * 17978 Δ2811::FRT carrying pBAD18Kan‐ori	This study
Δ*csuE*‐pABBR_MCS	*A. baumannii * 17978 Δ*csuE*::FRT carrying pABBR_MCS	This study
Δ2811::FRT‐c	*A. baumannii * 17978 Δ2811::FRT carrying pAT03	This study
Δ*csuE*::FRT‐c	*A. baumannii * 17978 Δ*csuE*::FRT carrying pAT04	This study
*Escherichia coli* DH5α		Lab stock
Plasmids		
pBAD18Kan‐ori	Kan^r^	Choi et al.
pKD4	Kan^r^	Lab stock
pABBR_MCS	Amp^r^	Tucker et al.
pAT01	pMMB67EH with Rec_Ab_ system	Tucker et al.
pAT02	pMMB67EH with FLP recombinase	Tucker et al.
pAT03	pBAD18Kan‐ori carrying A1S_2811	This study
pAT04	pABBR_MCS carrying *csuE*	This study

### 2.2 Identification of the operon containing A1S_2811

The organization of A1S_2811 and its surrounding genes suggests that five genes spanning A1S_2811 to A1S_2815 might belong to one operon. Primers to amplify the intergenic regions between these genes were designed (Table S1), and then synthesized by Sangon Biotech Co., (Shanghai, China). RNA was extracted from ATCC17978 and transcribed into cDNA. For the PCR amplifications, the extracted RNA and genomic DNA were set as the controls.

### 2.3 Creating gene knockouts with the Rec_Ab_ system

Recombination‐mediated chromosomal gene inactivation was performed as previously described (Tucker et al., 2014). A1S_2811 and A1S_2213 (*csuE*) were knocked out in ATCC17978. To knock out the entire 4,521 bp sequence of the A1S_2811 gene, we increased the length of the homologous regions to promote the recombination efficiency. By fusion PCR, we constructed a 2,047 bp DNA fragment containing 378 bases upstream and 330 bases downstream of the A1S_2811 coding sequence (CDS), flanking the kanamycin resistance cassette, which was amplified from the PKD4 plasmid. To knock out *csuE*, oligonucleotides containing 112 bases flanking the CDS of the *csuE* gene were synthesized by Sangon Biotech Co. *A. baumannii * carrying Rec_Ab_ on pMMB67EH (pAT01) was inoculated into LB media, which contained carbenicillin (100 μg/mL) to maintain the plasmid. Isopropyl β‐D‐1‐thiogalactopyranoside (IPTG) was added to a final concentration of 2 mmol/L, and the bacteria were grown at 37°C, 200 rpm for 3 h. After three washes with ice‐cold 10% glycerol, 100 μl of bacteria (~10^10^ bacteria) was mixed with 5 μg of the PCR products and then electroporated in a 2‐mm cuvette at 1.8 kV. After culturing in 4 ml of LB medium containing 2 mmol/L IPTG, the transformants were selected on kanamycin agar to identify colonies whose targeted genes were replaced by the kanamycin cassette. The selected colonies were verified by PCR and DNA sequencing, and then transferred to carbenicillin‐negative agar to cure the plasmid pAT01. Then, another plasmid containing the FLP/FRT recombinase system (pAT02) was electroporated into the selected colonies. After FLP/FRT recombination, the kanamycin resistance cassettes were replaced by the FLP recognition target (FRT) loci.

### 2.4 Complementation of mutants

Complementation vectors for the Δ2811::FRT and Δ*csuE*::FRT strains were constructed using the primer sets listed in Table S1. Plasmid pBAD18Kan‐ori (Choi, Slamti, Avci, Pier, & Maira‐Litran, 2009), provided by Professor Xilin Zhao of Xiamen University, China, was used to construct the A1S_2811 complementary strain. A1S_2811 gene was cloned into the multiple cloning site (MCS) in pBAD18Kan‐ori and electroporated into the Δ2811::FRT mutant. The *csuE* complementation was conducted by amplifying the full‐length gene with a primer set containing the Shine‐Dalgarno AGGAGG sequence (Table S1). Next, the PCR product was cloned into pABBR_MCS and electroporated into the Δ*csuE*::FRT mutant (Tucker et al., 2014).

### 2.5 Biofilm assay

Biofilm formation in the strains was tested as described previously (O'Toole et al., 1999; Tomaras, Dorsey, Edelmann, & Actis, 2003; Tucker et al., 2014). Briefly, a single colony was inoculated into 5 ml of LB broth followed by incubation overnight without shaking at 37°C. Then, the overnight culture was diluted 1:100 with LB. For each strain, 18 replicates of 100 μL aliquots of diluted culture were placed into each well of a polystyrene 96‐well cell culture plate and then grown without shaking for 24 h at 30°C (Tucker et al., 2014). Nine wells were used to determine the optical density (OD)_600_ to estimate the total cell biomass. The liquid from the other nine wells was aspirated carefully and the remaining biofilms were rinsed with distilled water. The biofilm walls were then stained with 0.1% crystal violet and solubilized with ethanol–acetone (O'Toole et al., 1999). The OD_580_ of the processed solution was determined and the OD_580_/OD_600_ ratio was used to measure the biofilm amounts (Tomaras et al., 2003). All assays were performed twice using fresh samples each time.

### 2.6 Motility assays

First, the strains were cultured on LB plates for two passages after recovery from the glycerol stocks. A single colony was then inoculated into 5 ml of LB broth and incubated for 24 h at 30°C without shaking prior to performing the assay. Next, the samples were adjusted to an OD_600_ of 0.6 with LB broth, and 2 μl of the bacterial culture was placed on the surface of the motility assay plates. Motility was investigated on motility plates after bacterial incubation at 37°C for 18 h. The motility plates were prepared with 10 g/L tryptone, 10 g/L NaCl and 5 g/L yeast extract, and the addition of 0.5% noble agar (Becton Dickinson, Sparks, MD, USA).

### 2.7 Transmission electron microscopy

Colonies on the motility plates were gently resuspended in 0.9 ml of HEPES buffer (0.85 ml of H_2_O plus 0.05 ml of 1 mol/L HEPES, pH 7.2), and the bacterial cells were then fixed by addition of 0.1 ml of glutaral (30%) (Wilharm, Piesker, Laue, & Skiebe, 2013). The samples were stained with 2% ammonium acetate and 2% ammonium molybdate (1:1) for 1.5 min. Images were obtained on an FEI Tecnai transmission electron microscope (FEI Company, Hillsboro, OR, USA).

### 2.8 Transcriptome analysis

Total RNA was isolated from ATCC17978 and the Δ2811::FRT mutant, both of which were previously grown on motility plates, using a Pure Link ^™^ RNA Mini Kit (Invitrogen, Carlsbad, CA, USA). The RNA concentration and quality of each sample were determined using a NanoDrop 2000 spectrophotometer (Thermo Fisher Scientific, Rockford, IL, USA). In total, 3 μg of RNA per sample was used as the input material for RNASeq library preparation. Strand‐specific transcriptome sequencing was performed by Novogene Bioinformatics Technology Co., Ltd. (Beijing, China). HTSeq v0.6.1 was used to count the number of reads mapped to each gene (Anders & Huber, 2010). The mapped fragments per kilo‐base of gene model per million reads associated with each gene was calculated based on the length of the gene and counts for the reads mapped to this gene (Trapnell, Pachter, & Salzberg, 2009). Prior to differential gene expression analysis, the read counts were adjusted using the edgeR program package using one scaling normalized factor for each sequenced library (Robinson, McCarthy, & Smyth, 2010). Differential expression analysis of two conditions was performed using the DEGSeq R package (1.20.0) (Wang, Feng, Wang, Wang, & Zhang, 2010). The *p* values were adjusted using the Benjamini and Hochberg method (Hochberg & Benjamini, 1990). A corrected *p* value (*q* value) of 0.005 and a log_2_ of 1 (fold change) was set as the threshold for significant transcription variation.

### 2.9 Reverse transcriptase‐PCR (RT‐PCR)

RT‐PCR was performed to verify parts of the transcriptome results (the primers used are shown in Table S1). All RNA samples were extracted from ATCC17978, Δ2811::FRT mutant and the complementation strain, which were previously grown on motility plates, using a Pure Link ^™^ RNA Mini Kit (Invitrogen, Carlsbad, CA, USA). The RT‐PCR protocol was conducted using SuperScript^®^ III Reverse Transcriptase (Invitrogen, USA). Reactions were performed in triplicate using 20 ng of cDNA template prepared from the appropriate cultures using SYBR^®^ Premix Ex *Taq* ^™^ II (Tli RNaseH Plus; Takara, Japan) on a LightCycler^®^ 480 II Real‐Time PCR System (Roche, Burgess Hill, UK) following the manufacturer‐supplied protocols. Expression was quantified relatively by comparison to that of 16S rRNA.

## 3 RESULTS

### 3.1 Identification of the operon containing A1S_2811

**Figure 1:**
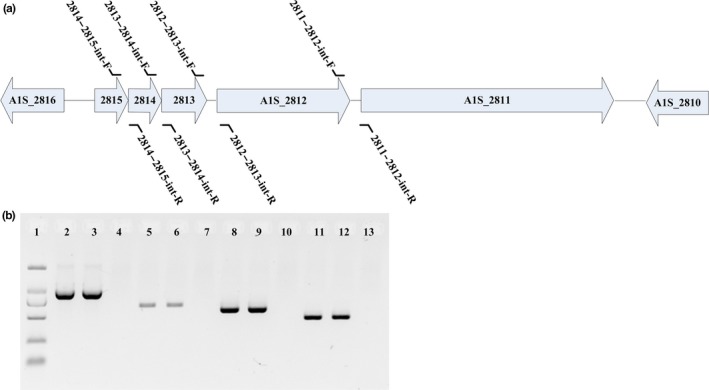
PCR confirmation of the A1S_2811‐2815 operon. (a) Organization of the A1S_2811‐2815 gene cluster. Arrows represent the transcriptional direction. The primers used are marked in their corresponding positions. (b) The primers were designed to detect the intergenic regions between 2811 and 2812 (lanes 2–4), 2812 and 2813 (lanes 5–7), 2813 and 2814 (lanes 8–10), and 2814 and 2815 (lanes 11–13). The templates used were cDNA (lanes 2, 5, 8, 11), genomic DNA from ATCC17978 (lanes 3, 6, 9, 12), and genomic RNA as a negative control (lanes 4, 7, 10, 13). Lane 1, 2,000‐kb DNA marker

The genome annotation of ATCC17978 suggests that A1S_2811 possesses four histidine‐containing phosphotransfer domains, a CheA‐like regulatory domain and a CheY‐like receiver domain at the C terminus (GenBank: NC_009085) (Smith et al., 2007). To investigate whether the five genes spanning A1S_2811 to A1S_2815 are parts of a single operon, primers were designed to amplify the intergenic regions between these genes. ATCC17978 cDNA was used as a PCR template, and genomic DNA was set as the control. The results demonstrated that A1S_2811 is cotranscribed with the following four upstream genes: A1S_2812 (*pilJ*), A1S_2813 *(pilI)*, A1S_2814 (*pilH*), and A1S_2815 (*pilG*) (Figure 1). Because A1S_2811 is the last gene in this five‐gene operon and the transcriptional direction of this operon is opposite to the adjacent gene A1S_2810, the probability of a polarity effect after A1S_2811 gene knockout should be relatively low.

### 3.2 Phenotypes of ATCC17978 and ∆2811::FRT

We applied a new recombination‐mediated knock‐out system (Rec_Ab_ system) to delete A1S_2811 from ATCC17978 and construct the Δ2811::FRT mutant. By PCR and sequencing tests, we confirmed that full‐length A1S_2811 was deleted and replaced by 91‐bp FRT loci. To investigate whether deleting A1S_2811 would affect the in vitro growth of *A. baumannii *, we tested the growth rates of the ∆2811::FRT and wild‐type (WT) strains in LB media (Mussi, Relling, Limansky, & Viale, 2007). There was no significant difference between them when grown on LB medium (Figure S1). Therefore, depleting A1S_2811 does not affect the in vitro growth of *A. baumannii * on LB medium.

**Figure 2:**
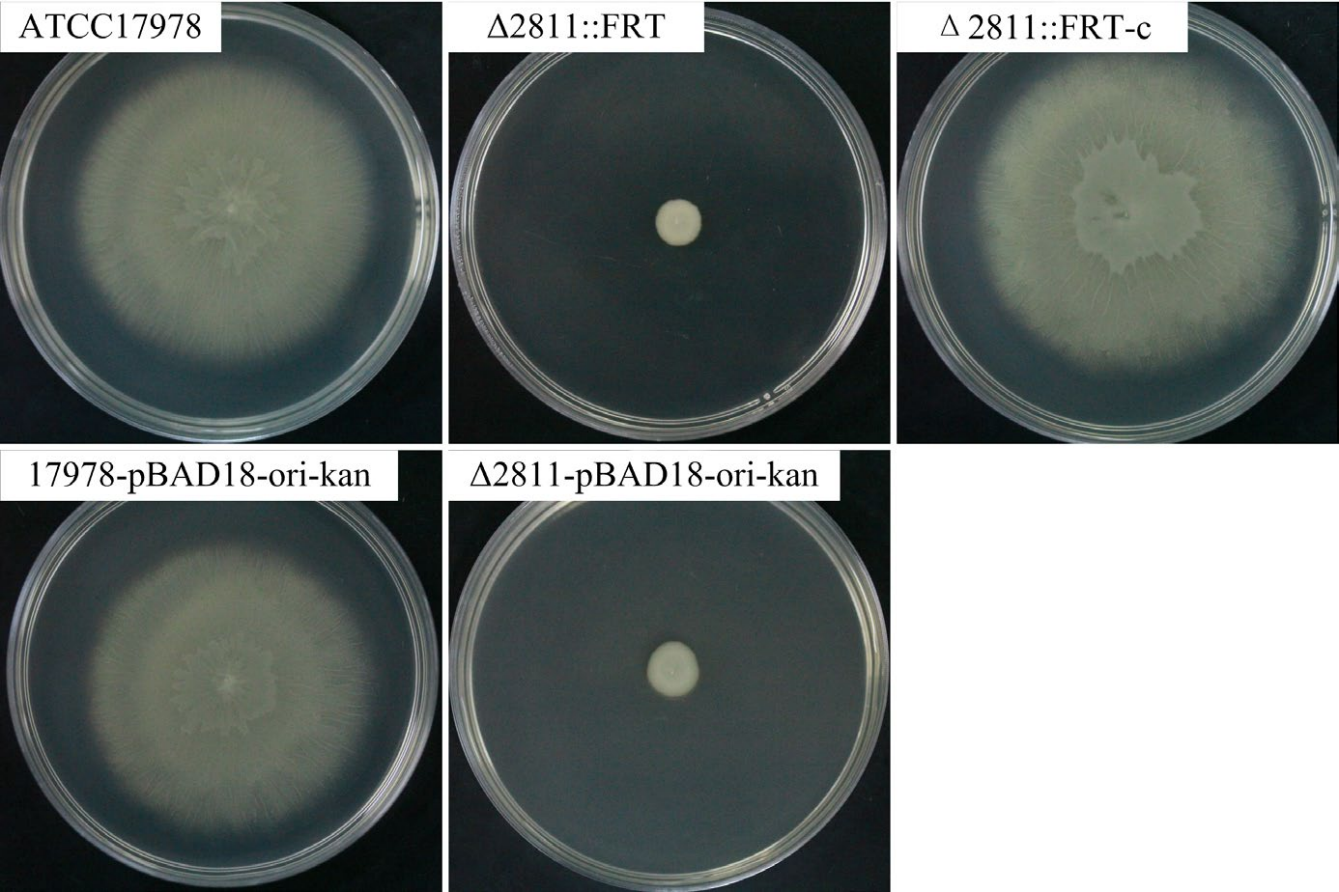
Surface motility assays for *A. baumannii* ATCC17978, Δ2811::FRT, Δ2811::FRT‐c, 17978‐pBAD18Kan‐ori, and Δ2811‐pBAD18Kan‐ori

We then tested the motility of ATCC17978 and Δ2811::FRT on motility plates. On the 0.5% noble agar motility plate, we found that ATCC17978 could not move at the interface between the bottom of the plate and the medium, and only surface motility on the motility plate was observed. Therefore, hereafter, when we talk about motility in this study, we are referring to “surface motility.” ATCC17978 formed round and translucent colonies with dense cells at the inoculation site on the motility plates. Peripheral cells radiated from the center and formed striations. The average colony diameter of ATCC17978 was 6.2 ± 0.8 cm (*n* = 5). In contrast, the Δ2811::FRT mutant lost its surface motility under the same conditions and formed smooth, thick, opaque round colonies with an average colony diameter of 0.7 ± 0.2 cm (*n* = 5). The surface motility was restored in the complementation strain (Figure 2).

**Figure 3:**
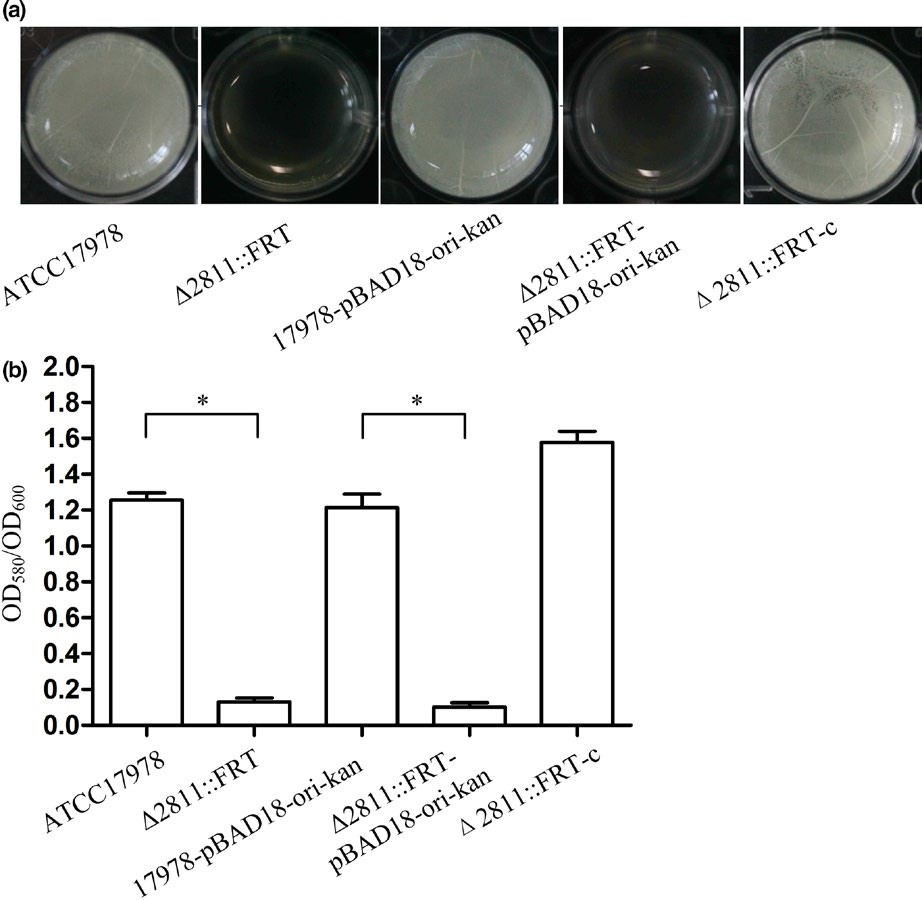
Biofilm formation in *A. baumannii* ATCC17978, Δ2811::FRT, Δ2811::FRT‐c, 17978‐pBAD18Kan‐ori, and Δ2811‐pBAD18Kan‐ori. (a) Biofilm formation at the gas–liquid interface. (b) Quantification of *A. baumannii * biofilm formation in polystyrene 96‐well cell culture plates. OD_580_/OD_600_ values were used to evaluate the biofilm amounts. Asterisks denote significant differences in biofilm formation (*t* test; *p < 0.0001; *n* = 9)

In the biofilm tests, a layer of pellicle at the gas–liquid interface of the culture was observed when *A. baumannii * ATCC17978 cultures were grown without shaking (Figure 3a). In the early phase of biofilm formation, particles were floating on the liquid surface, but when the biofilm started to develop, these floating particles attached to the wells. When incubated with shaking (200 rpm in an orbital shaker) at 37°C, ATCC17978 could barely form biofilm. The quantitative analysis of biofilm formation is shown in Figure 3b. The biofilm from Δ2811::FRT decreased significantly, whereas the complementation strain restored the ability to form biofilm.

**Figure 4:**
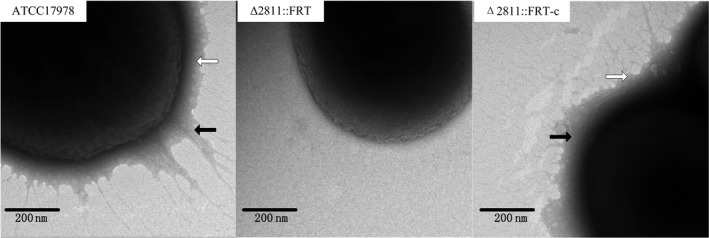
Transmission electron microscopy images of pilus‐like appendages and extrapolymeric substances (EPS) from *A. baumannii * strains grown on motility plates for 18 h. Black arrows specify pilus‐like appendages. White arrows specify EPS

To identify pilus‐like structures, cells from the *A. baumannii * strains on motility plates were examined by transmission electron microscopy. We observed a layer, most likely composed of extrapolymeric polysaccharides (EPS), surrounding cells from the WT strain. The pilus‐like structure on the cell surface almost disappeared in each Δ2811::FRT mutant, and EPS was rarely seen (Figure 4). The complementation strain restored the phenotype of ATCC17978.

### 3.3 Gene expression changes caused by deletion of A1S_2811

Transcriptome sequencing showed that after deletion of A1S_2811, the expression of 117 genes was significantly downregulated (log_2_.Fold_change <−1), whereas 80 genes were upregulated (log_2_.Fold_change >1) (Table S1 and S3). The genes with higher expression variations are listed in Tables 2 (log2.Fold_change <−2) and 3 (log2.Fold_change >2). The downregulated genes were enriched in the KEGG pathways of pantothenate and CoA biosynthesis, degradation of aromatic compounds, benzoate degradation, starch and sucrose metabolism, fatty acid biosynthesis, ethylbenzene degradation, fatty acid metabolism, and fluorobenzoate degradation. The most upregulated genes were A1S1792 (nucleoside‐diphosphate‐sugar epimerase), A1S_1791 (tartrate symporter MFS superfamily protein), and A1S_1805 (major facilitator superfamily transporter).

**Table 2:** Downregulated differentially expressed genes in the Δ2811::FRT mutant (log2.Fold_change <−2)

Gene_ID	Readcount_mu	Readcount_wt	log2.Fold_change	qvalue^a^	Gene_description
A1S_0113	2.9	2,952.1	−10	0	Acyl‐CoA dehydrogenase
A1S_0114	0	204.1	−9.6	9.90E‐36	Acyl carrier protein
A1S_0112	7.4	4,382.1	−9.2	0	Acyl‐CoA synthetase/AMP‐acid ligases II
A1S_0109	0	240.9	−8.8	2.70E‐46	Homoserine lactone synthase
A1S_0115	23	7,532.2	−8.4	0	Amino acid adenylation
A1S_0116	33.4	7,895.5	−7.9	0	RND superfamily transporter
A1S_1256	0	15.8	−5.9	1.40E‐04	Transcriptional regulator
A1S_2217	0	30.2	−5.8	6.70E‐08	Protein CsuA
A1S_2218	25.4	1,239.2	−5.6	3.30E‐290	Protein CsuA/B
A1S_0118	24.6	1,094.5	−5.5	6.20E‐256	Hypothetical protein
A1S_1292	14.3	530.1	−5.2	3.20E‐123	Signal peptide
A1S_0117	19.9	696	−5.1	3.80E‐161	Hypothetical protein
A1S_0119	5.3	151.5	−4.8	5.60E‐35	Phosphopantethiene‐protein transferase
A1S_2216	0	12.6	−4.6	1.10E‐03	Protein CsuB
A1S_2811	3.7	72.3	−4.3	2.80E‐16	Chemotactic signal transduction system component
A1S_1357	70.4	1,236.1	−4.1	5.50E‐262	Alanine racemase
A1S_0745	209.4	3,232	−3.9	0	Hypothetical protein
A1S_2213	3.4	47.7	−3.8	2.50E‐10	Protein CsuE
A1S_1294	12.1	147	−3.6	7.10E‐29	Hypothetical protein
A1S_1293	1.6	16.2	−3.4	7.90E‐04	Hypothetical protein
A1S_1509	7.4	77.6	−3.4	5.90E‐15	Pili assembly chaperone
A1S_2215	5.6	59.7	−3.4	7.80E‐12	Protein CsuC
A1S_2214	12.2	120.6	−3.3	2.60E‐22	Protein CsuD
A1S_0110	8.5	79	−3.2	1.30E‐14	Hypothetical protein
A1S_1295	48.4	438.9	−3.2	8.50E‐77	Hypothetical protein
A1S_1510	32.8	215.8	−2.7	1.00E‐32	Fimbrial protein
A1S_1233	116.2	703.8	−2.6	2.40E‐100	Hypothetical protein
A1S_2230	824.3	5,042.9	−2.6	0	Hypothetical protein
A1S_2511	33.1	193.8	−2.5	1.70E‐27	Phenylacetic acid degradation‐related protein
A1S_3445	21.2	118.4	−2.5	1.30E‐16	RND family cation/multidrug efflux pump
A1S_3447	26.5	149.7	−2.5	5.80E‐21	RND efflux transporter
A1S_3273	13	70.7	−2.4	4.00E‐10	Peptide signal
A1S_1078	5.6	24.8	−2.2	1.10E‐03	Hypothetical protein
A1S_1387	59.3	277.4	−2.2	2.20E‐33	Oxidoreductase
A1S_2074	22.5	106.9	−2.2	1.60E‐13	Hypothetical protein

a q value, corrected *p* value; smaller q values represent a more significant difference for the gene.

**Table 3:** Upregulated differentially expressed genes in the Δ2811::FRT mutant (log2.Fold_change >2)

Gene_ID	Readcount_mu	Readcount_wt	log2.Fold_change	qvalue^a^	Gene description
A1S_1792	99.5	6.2	4	1.70E‐21	Nucleoside‐diphosphate‐sugar epimerase
A1S_1791	106.7	10.4	3.4	2.30E‐20	Tartrate symporter MFS superfamily protein
A1S_1805	104	10.4	3.3	1.00E‐19	Major facilitator superfamily transporter
A1S_1794	69.4	7.6	3.2	5.80E‐13	Hypothetical protein
A1S_1790	54.3	7.3	2.9	1.50E‐09	6‐phosphogluconate dehydrogenase
A1S_1422	66.7	12.7	2.4	1.60E‐09	Triphosphoribosyl‐dephospho‐CoA synthase
A1S_1806	79.9	15.1	2.4	2.60E‐11	Senescence marker protein‐30
A1S_1426	62.5	14.9	2.1	1.10E‐07	Phosphoribosyl‐dephospho‐CoA transferase
A1S_0671	582.6	143.7	2	1.20E‐62	Protein tyrosine phosphatase
A1S_1795	86.6	21.45	2	6.00E‐10	Dihydroxy‐acid dehydratase
A1S_2169	987.4	243.7	2	1.00E‐105	Cytochrome o ubiquinol oxidase subunit IV

a *q* value, corrected *p* value; smaller *q* values represent a more significant difference for the gene.

**Figure 5:**
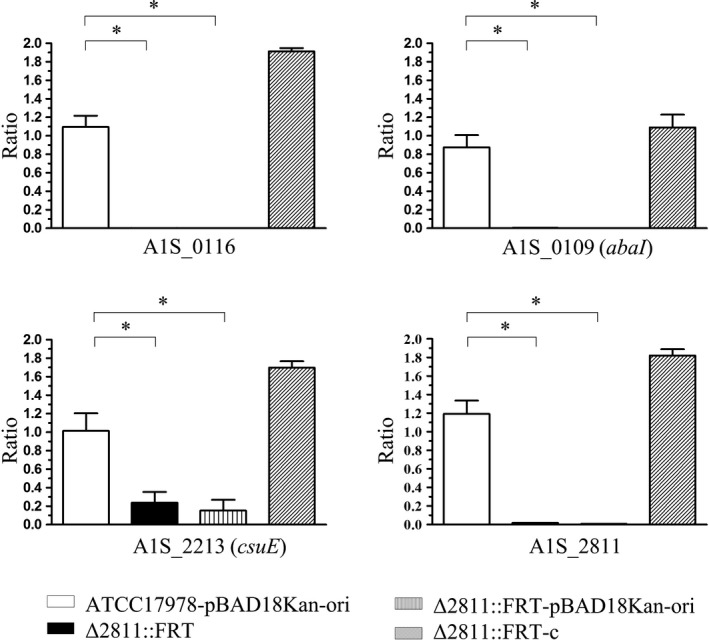
Transcriptome sequencing validation results for selected genes. Four genes (A1S_0116, A1S_0109 (*abaI*), A1S_2213 (*csuE*), and A1S_2811) were selected from Table 2. The expression ratio of each gene was calculated as the transcriptional level in each stain divided by the transcriptional level of the WT ATCC17978 strain. In addition to Δ2811::FRT, a WT strain carrying pBAD18Kan‐ori, a mutant carrying pBAD18Kan‐ori and the complementation strain were tested. Asterisks denote significant differences in the transcriptional levels (*t* test; *p < 0.0001; *n* = 3)

Within the downregulated genes, A1S_0109 (homoserine lactone synthase, *abaI*), A1S_0112–0119, and A1S_2213–2218 (*csuA/BABCDE operon*) might be associated with motility and biofilm formation in *A. baumannii * ATCC17978 according to the KEGG database on the KEGG website (Kanehisa, Sato, Kawashima, Furumichi, & Tanabe, 2016) and previous studies also (Clemmer et al., 2011; Giles, Stroeher, Eijkelkamp, & Brown, 2015). The high‐throughput transcriptome results were verified by RT‐PCR using four selected genes associated with motility and biofilm (Figure 5). All the genes we tested were downregulated in both Δ2811::FRT and Δ2811‐pBAD18Kan‐ori as expected, whereas the transcriptional levels of these genes were restored in the complementation strain (Figure 5). In addition to these four genes, we also compared the transcriptional profiles of 23 other genes related to type IV pili. The RT‐PCR results for these four genes verified the reliability of the RNASeq transcriptome results; the RT‐PCR results for the other tested genes are shown in Table S4.

### 3.4 Phenotypes of ATCC17978 and ∆ csuE::FRT

We found that the *csuA/BABCDE* operon is downregulated from the transcriptome analysis results of the A1S_2811 null mutant. Therefore, to investigate the role of the *csuA/BABCDE* operon in terms of motility and biofilm formation, we knocked out *csuE* (the last gene in the *csu* operon) while taking into consideration the necessity of avoiding the polarity effect, and constructed mutant ∆ *csuE*::FRT using the Rec_Ab_ system. By PCR and DNA sequencing, we confirmed that the full‐length *csuE* was deleted and replaced by 91‐bp FRT loci.

We tested the growth rates of the ∆ *csuE*::FRT and WT strain in LB medium (Mussi et al., 2007). There was no significant growth difference between them in this medium (Figure S2). Therefore, depleting *csuE* does not affect the in vitro growth of *A. baumannii * in the tested medium.

**Figure 6:**
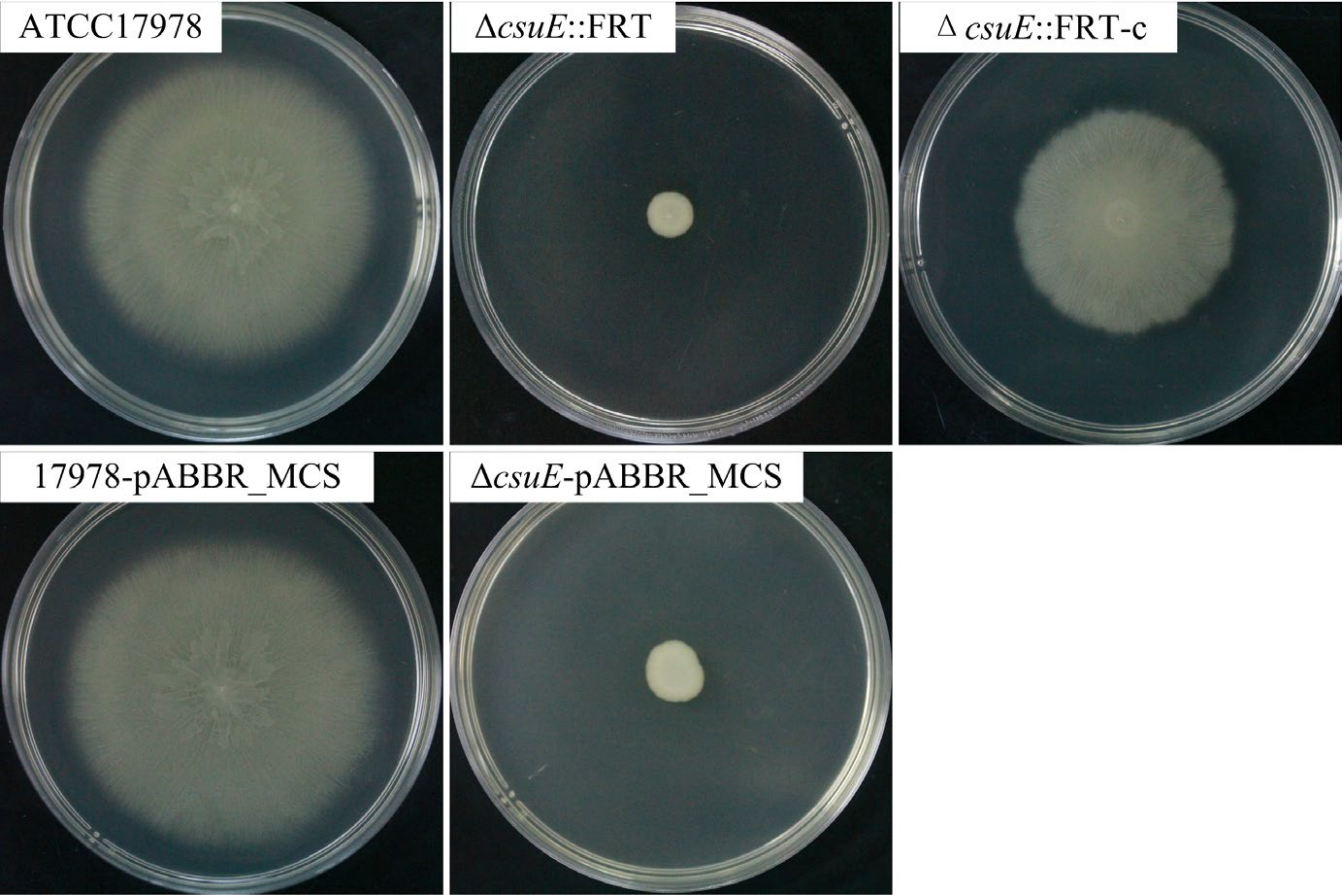
Surface motility assay for *A. baumannii* ATCC17978, Δ*csuE*::FRT, Δ*csuE*::FRT‐c, 17978‐pABBR_MCS, and Δ*csuE*‐pABBR_MCS

**Figure 7:**
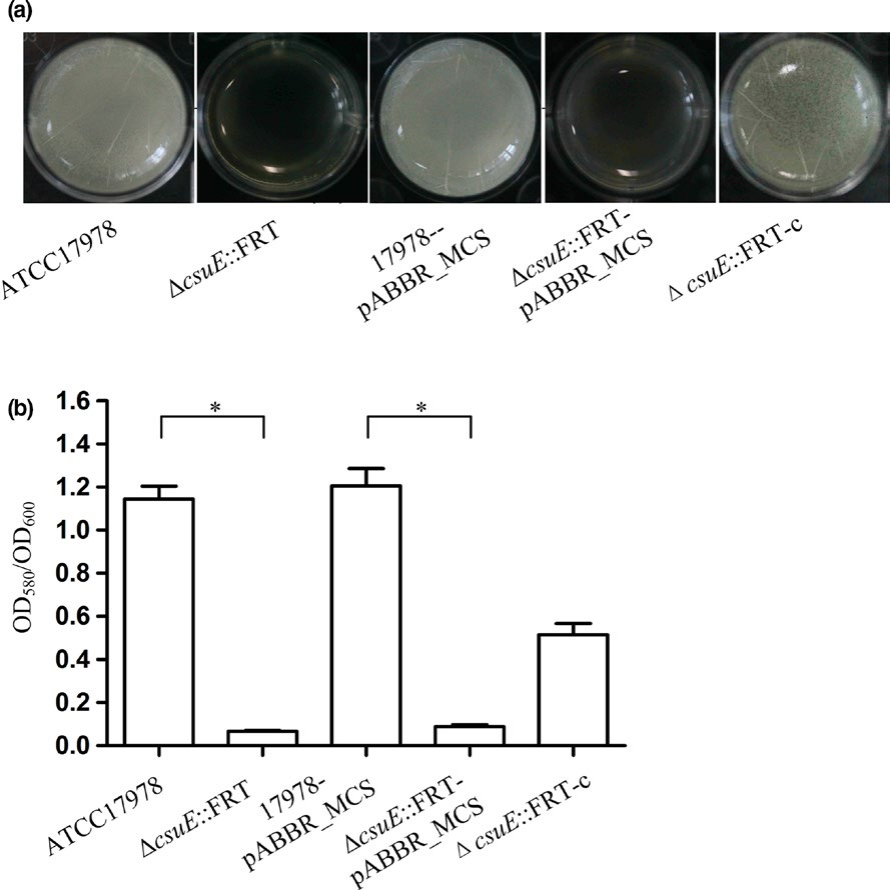
Biofilm formation in *A. baumannii* ATCC17978, Δ*csuE*::FRT, Δ*csuE*::FRT‐c, 17978‐pABBR_MCS, and Δ*csuE*‐pABBR_MCS. (a) Biofilm formation at the gas–liquid interface. (b) Quantification of *A. baumannii * biofilm formation in polystyrene 96‐well cell culture plates. OD_580_/OD_600_ values were used to evaluate the biofilm amounts. Asterisks denote significant differences in biofilm formation (*t* test; *p < 0.0001; *n* = 9)

Similar to ∆ *A1S_2811*::FRT, mutant ∆ *csuE*::FRT also showed reduced motility (Figure 6) and biofilm formation (Figure 7). Mutant ∆ *csuE*::FRT formed smooth, thick, opaque round colonies on motility plates with an average colony diameter of 0.7 ± 0.2 cm (*n* = 5) versus 6.2 ± 0.8 cm (*n* = 5) for ATCC17978. The complementation strain had biofilm formation and motility restored.

### 3.5 Supplementation with synthetic *N*‐(3‐oxodecanoyl) homoserine‐l‐lactone restored the phenotype of Δ2811::FRT

**Figure 8:**
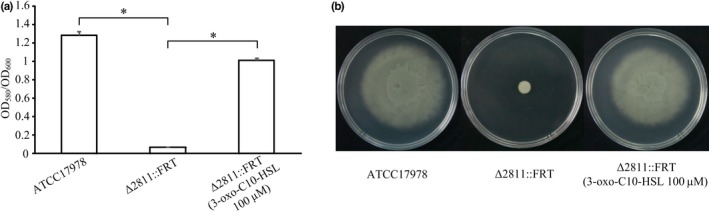
Supplementation with synthetic 3‐oxo‐C10‐HSL restores the phenotype of the Δ2811::FRT mutant. (a) Quantification of biofilm formation in ATCC17978 and Δ2811::FRT (plus or minus 3‐oxo‐C10‐HSL). OD_580_/OD_600_ values were used to evaluate the biofilm amounts. Asterisks denote significant differences in biofilm formation (*t* test; *p < 0.0001; *n* = 9). (b) Surface motility of ATCC17978 and Δ2811::FRT (plus or minus 3‐oxo‐C10‐HSL) on 0.5% noble agar motility plates

Among the genes in Table 2, A1S_0109 (*abaI*) was reported as being the only autoinducer synthase encoded in the *A. baumannii * genome (Niu, Clemmer, Bonomo, & Rather, 2008). To investigate the possible role of bacterial quorum sensing and the regulation of A1S‐2811 in ATCC17978, we supplied 100 μM synthetic *N*‐(3‐Oxodecanoyl)‐l‐homoserine lactone (3‐oxo‐C10 HSL) to the mutant ∆2811::FRT when performing biofilm and motility tests. As shown in Figure 8, the motility and biofilm defects were rescued completely with 3‐oxo‐C10 HSL.

## 4 DISCUSSION

In this study, we have shown that deleting A1S_2811 decreased the surface motility and biofilm formation of *A. baumannii * ATCC17978. Biofilm, a structure of connected cells surrounded by a matrix of extracellular polysaccharides (Moonmangmee et al., 2002; Yamamoto, Arai, Ishii, & Igarashi, 2012), is associated with multidrug resistance in *A. baumannii * (Badave & Kulkarni, 2015; Rao et al., 2008). The pellicle is a special form of biofilm localized in the air–liquid interface (Branda, Vik, Friedman, & Kolter, 2005). In our study, we found that the motility and biofilm formation phenotypes of ATCC17978 were closely related. Culture liquid incubated at 30°C for about 24 h without shaking produced pellicle biofilms and the bacteria showed a motility phenotype on motility plates. When ATCC17978 was preincubated with shaking, no gas–liquid interface biofilm formed and the strain showed no motility on the motility plates. We have no explanation for this as yet. A previous study also found that surface film‐forming strains were motile (Giles et al., 2015). In contrast, another study found that clinical respiratory isolates frequently formed more biofilm and were less motile than nonclinical strains (Vijayakumar et al., 2016). The association between motility and biofilm formation in *A. baumannii * remains ambiguous; this lack of clarity may be related to different genetic backgrounds in the strains of this species and requires further investigation. Notably, although both ∆2811::FRT and ∆*csuE*::FRT were nonmotile on the motility plates (Figures 2 and 6), they might not be completely defective in motility. When grown on plates with only 0.3% agar, they also formed large colonies. However, their colonies were far smaller than those of the WT strain. This implies that bacterial regulatory mechanisms and mechanisms involved in motility are complex.

A1S_2811 in ATCC17978 possesses potential phosphorylation sites and a CheY‐like receiver domain, suggesting that it is a hypothetical hybrid TCS (Burbulys, Trach & Hoch, 1991; Capra & Laub, 2012). Through PCR, we confirmed that A1S_2811 is cotranscribed with four other upstream genes, namely, A1S_2812 (*pilJ*), A1S_2813 *(pilI)*, A1S_2814 (*pilH*), and A1S_2815 (*pilG*). Homologs of these genes have been verified as being associated with motility in various bacterial species (Bertrand et al., 2010; Chung et al., 2001; Gooderham & Hancock, 2009; Whitchurch et al., 2004). In *P. aeruginosa, chpA* (an A1S_2811 homolog) is downstream of *pilK*,* pilJ, pilI pilH*, and *pilG*. Although a *pilK* homolog is not found in the corresponding location in *A. baumannii,* the structure of these two operons is almost identical. *chpA* was found to be associated with type IV pili assembly and/or retraction as well as expression of the pilin subunit gene *pilA* (Whitchurch et al., 2004). ChpA functions upstream of PilH and PilT and the histidine kinase domain of ChpA, and the phosphoacceptor sites of both PilG and PilH are required for type IV pili function (Bertrand et al., 2010).

Previous studies have also shown that motility in *A. baumannii * is associated with type IV pili (Clemmer et al., 2011; Harding et al., 2013). However, in our study expression of the type IV pili genes related to twitching motility and type IV pilus assembly were not affected by deleting A1S_2811, as confirmed by RT‐PCR (Table S4); these genes include A1S_2812 (*pilJ*), A1S_2813 (*pilI*),A1S_2814 (*pilH*), A1S_2815 (*pilG*), A1S_0232(*pilR*), A1S_0234 (*pilR*), A1S_0235 (*pilS*), A1S_3177 (*pilA*), A1S_0327 (*pilD*), A1S_0328 (*pilC*), A1S_0329 (*pilB*), A1S_0896 (*pilU*), A1S_0897 (*pilT*), A1S_0500 (*pilF*), A1S_3191 (*pilQ*), A1S_3192 (*pilP*), A1S_3193 (*pilO*), A1S_3194 (*pilN*), A1S_3195 (*pilM)*, A1S_1559 (*pilZ*), A1S_3165 (*pilE*), A1S_3166 (*pilE*), A1S_3167 (*pilY1*), and A1S_3168 (*pilW*). Interestingly, although A1S_2811 was cotranscribed with A1S_2812 (*pilJ*), A1S_2813 (*pilI*), A1S_2814 (*pilH*), and A1S_2815 (*pilG*), it does not regulate their transcription.

As we did not observe significant transcriptional variations for type IV pili in the Δ2811::FRT mutant in this study, we speculate that A1S_2811‐related surface motility and biofilm formation might be independent of type IV pili. Our study suggests that A1S_2811‐mediated surface motility and biofilm formation might be associated with chaperone/usher (CU) pili instead. CU pilus is a type of nonflagellar appendage assembled on an outer membrane assembly platform called the usher where the periplasmic chaperone‐bound pilus subunits are polymerized in an orderly fashion (Sauer, Remaut, Hultgren, & Waksman, 2004). CU pili can be found in diverse gram‐negative bacteria, including important human and animal pathogens (Sauer et al., 2004). In the list of genes downregulated in the absence of A1S_2811, the *csuA/BABCDE* operon, which is responsible for CU pili assembly (Nait Chabane et al., 2014; Tomaras et al., 2003), is significantly downregulated (Table 2). The *csuA/BABCDE* operon is required for biofilm formation on solid surfaces, and knocking‐out *csuE* in ATCC19606 resulted in a biofilm‐deficient phenotype and pili disappearance (Tomaras et al., 2003, 2008). Little is known about the relationship between the *csuA/BABCDE* operon and motility in *A. baumannii *, except that one study found that deleting *csuD* in the *A. baumannii * M2 strain did not affect its motility (Clemmer et al., 2011), but as reported before for *csuA* mutant and *csuE* mutant (Tomaras et al., 2003), the Δ*csuD* mutant exhibits a biofilm formation defect (Harding et al., 2013). To further investigate the role of CU pili in biofilm formation and motility, we constructed another mutant, Δ*csuE*::FRT, and found that *csuE* was associated with motility and biofilm formation in ATCC17978. Differences in the results we obtained might be related to the different strains we used and the different genes in the *csuA/BABCDE* operon we investigated.

Transcriptome analysis of the Δ2811 null mutant in comparison with ATCC17978 also showed that the transcriptional level of another operon (A1S_0112–0119) decreased significantly. This operon is annotated as being responsible for the nonribosomal production of a lipopeptide that possibly acts as a surfactant to aid motility. Previous studies have shown that the A1S_0112–0119 operon is essential for pellicle formation (Giles et al., 2015) and motility (Clemmer et al., 2011) in *A. baumannii *. The motility and pellicle phenotypes of *A. baumannii * might be linked via the expression of cAMP and the A1S_0112–0119 operon (Giles et al., 2015).

After knocking out A1S_2811, the transcriptional level of A1S_0109 (*abaI*) also decreased significantly; this gene is annotated as the only autoinducer synthase encoded in the *A. baumannii * genome (Niu et al., 2008). An *abaI*::Km mutant failed to produce any detectable AHL (*N*‐acylhomoserine lactone) signals and was impaired in biofilm development in the *A. baumannii * M2 strain (Niu et al., 2008). Additionally, Luo et al. (2015) reported that non‐native acyl‐homoserine lactone could enhance pili assembly and biofilm formation in *A. baumannii * ATCC19606. In our study, the Δ2811::FRT phenotype was rescuable by supplementation with synthetic 3‐oxo‐C10 HSL (one of the quorum‐sensing AHLs). Collectively, these studies confirm that autoinducer‐dependent quorum sensing plays a vital role in regulating motility and biofilm formation in *A. baumannii *. The A1S_0112‐0119 operon was previously reported to be activated by quorum‐sensing signals A1S_0109 (*abaI*) (Clemmer et al., 2011; Giles et al., 2015). On the basis of the published scientific literature and our own results, we speculate that A1S_2811 is part of a TCS that regulates the A1S_0112‐0119 operon via the AbaI‐dependent quorum‐sensing pathway in ATCC17978.

In this study, we confirmed that A1S_2811, a CheA/Y‐like hybrid, two‐component regulator in *A. baumannii * ATCC17978, is involved in this bacterium's surface motility and biofilm formation phenotypes. The motility of ATCC17978 seemed to be not associated with the retraction of type IV pili, but was instead related to CU pili, a lipopeptide encoded by the A1S_0112–0119 operon and to the AbaI‐dependent quorum‐sensing pathway. A1S_2811 might regulate surface motility and biofilm formation via regulating the *csuA/BABCDE* operon associated with CU pili and the AbaI‐dependent quorum‐sensing pathway‐associated A1S_0112‐0119 operon. However, the detailed regulation networks governing the exact mechanisms of interaction between the *csuA/BABCDE* operon, the A1S_0112–0119 operon and A1S_0109 (*abaI*) await further investigation.

## CONFLICTS OF INTEREST

There are no conflicts of interest.
