# Supplementary material for: A1S_2811, a CheA/Y‐like hybrid two‐component regulator from Acinetobacter baumannii ATCC17978, is involved in surface motility and biofilm formation in this bacterium
Source: Microbiologyopen. 2017 Jul 17;6(5):e00510. doi: 10.1002/mbo3.510 (PMC5635159; doi:10.1002/mbo3.510)
Supplement: Supplementary file 1 [file MBO3-6-na-s001.pdf]

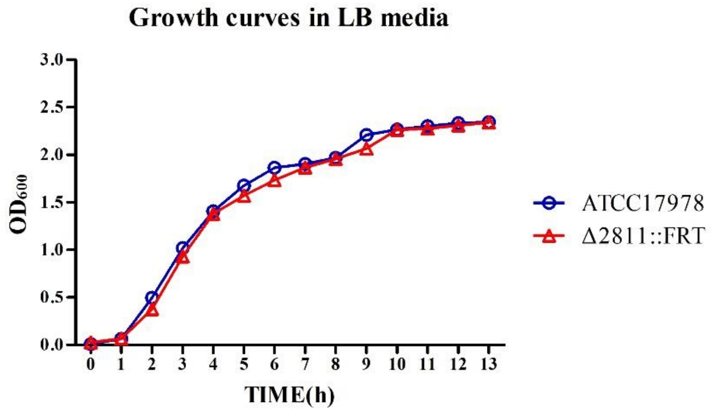

Figure S1. ATCC17978 and mutant Δ2811::FRT growth curves in LB broth.

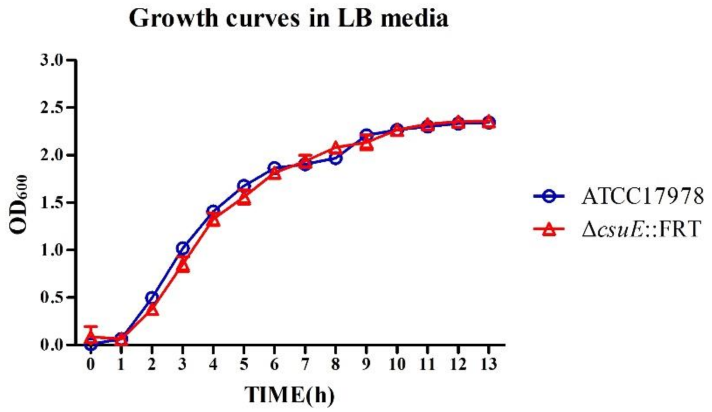

Figure S2. ATCC17978 and mutant Δ*csuE*::FRT growth curves in LB broth.
