# Supplementary material for: A1S_2811, a CheA/Y‐like hybrid two‐component regulator from Acinetobacter baumannii ATCC17978, is involved in surface motility and biofilm formation in this bacterium
Source: Microbiologyopen. 2017 Jul 17;6(5):e00510. doi: 10.1002/mbo3.510 (PMC5635159; doi:10.1002/mbo3.510)
Supplement: Supplementary file 2 [file MBO3-6-na-s002.docx]

Table S1. Oligonucleotides used in this study

| Application | Primer | Sequence (5’ to 3’) |
| --- | --- | --- |
| primers for operon confirmation | | |
|  | 2811-2812-int-F | ACATCGTATCGCTCATTA |
|  | 2811-2812-int-R | GTTGTTGTAGTTGTTGGC |
|  | 2812-2813-int-F | TATGTGGGGTTGGTTGTG |
|  | 2812-2813-int-R | TTTGATATTCATCGGTCG |
|  | 2813-2814-int-F | CCAGATTACCCGTGATGA |
|  | 2813-2814-int-R | CACAACCAACCCCACATA |
|  | 2814-2815-int-F | TTCCCGTTATCATGCTCT |
|  | 2814-2815-int-R | CCATACTCGGTCAGTTGC |
| primers for recombination | | |
|  | 2811_h_kana_F | TCAGGGGGTAAGCAAAAGAAGCCTTTGCTAGCGATTGTGTAGGCTGGAGCTGCTTCG |
|  | 2811_h_kana_R | AGCTCTTGTTGGTCCATATTAGCGATCAATGATGTATTTACACTACCTTCATATGAATATCCTCCTTAGTTCCTATTCCG |
|  | 2811_up_h_F | GTTTGATACTGCTCGCTCCGT |
|  | 2811_up_h_R | CTTCTTTTGCTTACCCCCTGA |
|  | 2811_down_h_F | TAATATGGACCAACAAGAGCT |
|  | 2811_down_h_F | AATACGCAGACGAGTAGAAAC |
|  | 2213_h_kana_F | AAGCCAAGCAGCAGATTGTGGTGGTTAAACCTGTTACTTGTCACGAGGTATCTTTGCCATGAATATAAAAACAAAAAAATTACTCAGACATTTATGTATATTCTCTGGACTGAGCGATTGTGTAGGCTGG |
|  | 2213_h_kana_R | TAAAAAGATAAAAATTAAAATTACCAAAGATGATGATCAGTTAGAAATTGAATAGTTTGGTTAAAGATAAAAGCCCTTTAACTGAGCTTTTATCTTCTATATAAAACAGCTTCATATGAATATCCTCCTT |
| primers for complement | | |
|  | 2811_com_F | GGCTAGCGAATTCGAGCTCGATGAAAGAAATATTAAAAAC |
|  | 2811_com_R | AACAGCCAAGCTTGCATGCCAGCTCTTGTTGGTCCATATTA |
|  | 2213_com_F | TCCCCCCGGGAGGAGGAAACGATGTTGACAGGAAATTTGGCTCATG |
|  | 2213_com_R | AACTGCAGTTAAAACTCGACTTGTACCGTGACCGTATC |
| RT-PCR primers | | |
|  | Ab_16S_F | GGAGGAAGGTGGGGATGACG |
|  | Ab_16S_R | ATGGTGTGACGGGCGGTGTG |
|  | A1S_0109_F | TTTCCCCAACTTCTC |
|  | A1S_0109_R | ACACAGCCTGACTGC |
|  | A1S_0116_F | TGGTGAGCCGACTGATTT |
|  | A1S_0116_R | TTTCTTTGATGCGGTGAT |
|  | A1S_0232_F | TACATCCTGCTCCTCTAA |
|  | A1S_0232_R | CTAAGCCTTCACTTGGTA |
|  | A1S_0234_F | AAATGACACTTGCACGAA |
|  | A1S_0234_R | GTCGGTTAAACACGCATC |
|  | A1S_0235_F | CCTGTTCACTCTTGGGTA |
|  | A1S_0235_R | AGCGGTCACCTTCTTTTA |
|  | A1S_0327_F | CACCCAACTCAGCCATTT |
|  | A1S_0327_R | CCGTAGCCCATACCTTCT |
|  | A1S_0328_F | CTCGAAACGATGCTGGAC |
|  | A1S_0328_R | AATAACTGTTGCTGGGTA |
|  | A1S_0329_F | CCTACCGCTCAAGACGAT |
|  | A1S_0329_R | TCTGAAGCACCCATACGA |
|  | A1S_0500_F | AAGCGGAACATTACTTTA |
|  | A1S_0500_R | AATTGCTCAATAGCATCA |
|  | A1S_0896_F | GGTGAGATCCGTTCTCGT |
|  | A1S_0896_R | CTTGGTTGGCGTTGTTGG |
|  | A1S_0897_F | TATCCTCGTGGCATTGTA |
|  | A1S_0897_R | ATGGTCATAGCGGTTTTC |
|  | A1S_1559_F | AATCACAAAAAATTCCTT |
|  | A1S_1559_R | AAACTCCTTTATCACCAC |
|  | A1S_2213_F | CTGGGCAAAGTGTATCGC |
|  | A1S_2213_R | TCAAAGAAGGCACCGATT |
|  | A1S_2811_F | TAGAAATGGCTGAGTCGC |
|  | A1S_2811_R | TGCACCACCTTTCAAACT |
|  | A1S_2812_F | TCTATGGCTGGTGAGGCT |
|  | A1S_2812_R | TTCGATCTGTTTGGTTGC |
|  | A1S_2813_F | ATACTTCGTCGCACCACT |
|  | A1S_2813_R | GAGCCAAATCAGATACCG |
|  | A1S_2814_F | CGGTGGTGATTGTGAGTA |
|  | A1S_2814_R | TTCTTCGATTGGCTTGAT |
|  | A1S_2815_F | TACTGCTGTCGATGGATT |
|  | A1S_2815_R | TTGATAACCGTCTAAACG |
|  | A1S_3165_F | ACACCAATCGCTAATACT |
|  | A1S_3165_R | CAATCTGTAGCACCTTTT |
|  | A1S_3166_F | CTGCAAATGGGCAAACAT |
|  | A1S_3166_R | TGACAACGCAAACCTGAA |
|  | A1S_3167_F | ACAGTAACGATCCACGAT |
|  | A1S_3167_R | GTCATACCTTCACGACCT |
|  | A1S_3168_F | CAGGGCAAATTATACTGC |
|  | A1S_3168_R | CCACCCGATACTGAGGAA |
|  | A1S_3177_F | ACACTGCTCGTTCTCAAA |
|  | A1S_3177_R | GCTGCGGGGTTATTATTC |
|  | A1S_3191_F | AACATACTTCTGGTGGAG |
|  | A1S_3191_R | ATCTTGAGTAATAGTGCC |
|  | A1S_3192_F | ATCAGCCGCCACTCCCTA |
|  | A1S_3192_R | ACCCGTTTCCCAGCCATA |
|  | A1S_3193_F | TGAGATTCCGAGTTTAGT |
|  | A1S_3193_R | AATTTCCTGCTTTACTTC |
|  | A1S_3194_F | TAAACGGCTTACAGGAAC |
|  | A1S_3194_R | AAATACATAGCTGGTGGT |
|  | A1S_3195_F | GTCACGATTGCCAACCCA |
|  | A1S_3195_R | AAGCCACATGCCACCATT |

Table S2 A list of differentially expressed genes that are down-regulated in mutant Δ2811::FRT (log2.Fold_change<-1)

| Gene_ID | Readcount  _mu | Readcount  _wt | log2.Fold  _change | qvalue^a^ | Gene_description |
| --- | --- | --- | --- | --- | --- |
| A1S_0113 | 2.9 | 2952.1 | -10 | 0.00E+00 | acyl-CoA dehydrogenase |
| A1S_0114 | 0 | 204.1 | -9.6 | 9.90E-36 | acyl carrier protein |
| A1S_0112 | 7.4 | 4382.1 | -9.2 | 0.00E+00 | acyl-CoA synthetase/AMP-acid ligases II |
| A1S_0109 | 0 | 240.9 | -8.8 | 2.70E-46 | homoserine lactone synthase |
| A1S_0115 | 23 | 7532.2 | -8.4 | 0.00E+00 | amino acid adenylation |
| A1S_0116 | 33.4 | 7895.5 | -7.9 | 0.00E+00 | RND superfamily transporter |
| A1S_1256 | 0 | 15.8 | -5.9 | 1.40E-04 | transcriptional regulator |
| A1S_2217 | 0 | 30.2 | -5.8 | 6.70E-08 | protein CsuA |
| A1S_2218 | 25.4 | 1239.2 | -5.6 | 3.30E-290 | protein CsuA/B |
| A1S_0118 | 24.6 | 1094.5 | -5.5 | 6.20E-256 | hypothetical protein |
| A1S_1292 | 14.3 | 530.1 | -5.2 | 3.20E-123 | signal peptide |
| A1S_0117 | 19.9 | 696 | -5.1 | 3.80E-161 | hypothetical protein |
| A1S_0119 | 5.3 | 151.5 | -4.8 | 5.60E-35 | phosphopantethiene-protein transferase |
| A1S_2216 | 0 | 12.6 | -4.6 | 1.10E-03 | protein CsuB |
| A1S_2811 | 3.7 | 72.3 | -4.3 | 2.80E-16 | chemotactic signal transduction system component |
| A1S_1357 | 70.4 | 1236.1 | -4.1 | 5.50E-262 | alanine racemase |
| A1S_0745 | 209.4 | 3232 | -3.9 | 0.00E+00 | hypothetical protein |
| A1S_2213 | 3.4 | 47.7 | -3.8 | 2.50E-10 | protein CsuE |
| A1S_1294 | 12.2 | 147 | -3.6 | 7.10E-29 | hypothetical protein |
| A1S_1293 | 1.6 | 16.2 | -3.4 | 7.90E-04 | hypothetical protein |
| A1S_1509 | 7.4 | 77.6 | -3.4 | 5.90E-15 | pili assembly chaperone |
| A1S_2215 | 5.6 | 59.7 | -3.4 | 7.80E-12 | protein CsuC |
| A1S_2214 | 12.2 | 120.6 | -3.3 | 2.60E-22 | protein CsuD |
| A1S_0110 | 8.5 | 79 | -3.2 | 1.30E-14 | hypothetical protein |
| A1S_1295 | 48.4 | 438.9 | -3.2 | 8.50E-77 | hypothetical protein |
| A1S_1510 | 32.8 | 215.8 | -2.7 | 1.00E-32 | fimbrial protein |
| A1S_1233 | 116.2 | 703.8 | -2.6 | 2.40E-100 | hypothetical protein |
| A1S_2230 | 824.3 | 5042.9 | -2.6 | 0.00E+00 | hypothetical protein |
| A1S_2511 | 33.1 | 193.8 | -2.5 | 1.70E-27 | phenylacetic acid degradation-related protein |
| A1S_3445 | 21.2 | 118.4 | -2.5 | 1.30E-16 | RND family cation/multidrug efflux pump |
| A1S_3447 | 26.5 | 149.7 | -2.5 | 5.80E-21 | RND efflux transporter |
| A1S_3273 | 13 | 70.7 | -2.4 | 4.00E-10 | peptide signal |
| A1S_1078 | 5.6 | 24.8 | -2.2 | 1.10E-03 | hypothetical protein |
| A1S_1387 | 59.3 | 277.4 | -2.2 | 2.20E-33 | oxidoreductase |
| A1S_2074 | 22.5 | 106.9 | -2.2 | 1.60E-13 | hypothetical protein |
| A1S_1824 | 12.2 | 48.1 | -2 | 9.20E-06 | hypothetical protein |
| A1S_2000 | 31.5 | 124.7 | -2 | 1.80E-13 | hypothetical protein |
| A1S_2648 | 63.5 | 245.8 | -2 | 3.70E-25 | hypothetical protein |
| A1S_1079 | 40 | 144.8 | -1.9 | 3.10E-14 | dichlorophenol hydroxylase |
| A1S_1366 | 39.4 | 146.2 | -1.9 | 1.10E-14 | amino acid transporter LysE |
| A1S_1438 | 13.2 | 49 | -1.9 | 1.50E-05 | coenzyme F420-dependent N5N10-methylene tetrahydromethanopterin reductase |
| A1S_3446 | 41 | 150.8 | -1.9 | 5.40E-15 | RND family cation/multidrug efflux pump |
| A1S_1508 | 10.1 | 36.2 | -1.8 | 3.10E-04 | fimbrial biogenesis outer membrane usher protein |
| A1S_1829 | 10.6 | 36.1 | -1.8 | 5.10E-04 | hypothetical protein |
| A1S_1832 | 32.3 | 115.6 | -1.8 | 2.00E-11 | oxidoreductase FMN-binding |
| A1S_2228 | 34.1 | 115.5 | -1.8 | 9.30E-11 | hypothetical protein |
| A1S_2643 | 16.9 | 58.8 | -1.8 | 4.20E-06 | short chain dehydrogenase/reductase family oxidoreductase |
| A1S_2649 | 32 | 113.8 | -1.8 | 3.60E-11 | regulatory protein |
| A1S_3362 | 31.8 | 112.4 | -1.8 | 5.40E-11 | hypothetical protein |
| A1S_0058 | 132.4 | 435.1 | -1.7 | 5.50E-37 | glycosyltransferase |
| A1S_0157 | 107.5 | 345.6 | -1.7 | 9.20E-29 | hypothetical protein |
| A1S_0804 | 259.9 | 828.6 | -1.7 | 2.40E-67 | trehalose-6-phosphate phophatase |
| A1S_1439 | 9.5 | 32.1 | -1.7 | 1.20E-03 | coenzyme F420-dependent N5N10-methylene tetrahydromethanopterin reductase |
| A1S_1498 | 270 | 857.2 | -1.7 | 2.70E-69 | TetR family transcriptional regulator |
| A1S_1834 | 15.6 | 49.7 | -1.7 | 7.00E-05 | hypothetical protein |
| A1S_0059 | 159.4 | 481.5 | -1.6 | 7.80E-37 | glycosyltransferase |
| A1S_0173 | 12.7 | 37.3 | -1.6 | 1.40E-03 | transcription regulator protein |
| A1S_0617 | 155.9 | 460.4 | -1.6 | 3.30E-34 | hypothetical protein |
| A1S_2161 | 59.3 | 178.8 | -1.6 | 3.60E-14 | hemin storage signal peptide protein |
| A1S_0087 | 310.2 | 876.7 | -1.5 | 9.70E-61 | short-chain dehydrogenase |
| A1S_0267 | 24.1 | 68.1 | -1.5 | 1.50E-05 | thioesterase |
| A1S_0302 | 41 | 119.6 | -1.5 | 2.20E-09 | hypothetical protein |
| A1S_1318 | 74.1 | 203.8 | -1.5 | 3.60E-14 | N-acetyltransferase GCN5 |
| A1S_1383 | 1212.6 | 3536.9 | -1.5 | 4.90E-255 | surface antigen |
| A1S_1851 | 40.5 | 110.7 | -1.5 | 4.70E-08 | penicillin G amidase |
| A1S_2090 | 35.5 | 103.1 | -1.5 | 3.60E-08 | hypothetical protein |
| A1S_2091 | 237.7 | 659.8 | -1.5 | 1.10E-44 | hypothetical protein |
| A1S_2227 | 28.6 | 83 | -1.5 | 8.90E-07 | methyltransferase |
| A1S_2229 | 81.8 | 230 | -1.5 | 2.30E-16 | acyl-CoA dehydrogenase-related protein |
| A1S_2509 | 97.1 | 268.6 | -1.5 | 1.50E-18 | chaperone |
| A1S_1385 | 1153.3 | 3012.8 | -1.4 | 1.80E-184 | hypothetical protein |
| A1S_1497 | 21.7 | 58.3 | -1.4 | 1.40E-04 | acyltransferase |
| A1S_1698 | 105.9 | 278.3 | -1.4 | 8.50E-18 | lipoyl synthase |
| A1S_1811 | 38.9 | 105.8 | -1.4 | 1.10E-07 | hypothetical protein |
| A1S_2160 | 57.4 | 153.5 | -1.4 | 1.80E-10 | hemin storage system HmsR protein |
| A1S_2162 | 133.9 | 344.7 | -1.4 | 3.70E-21 | hypothetical protein |
| A1S_2695 | 44.7 | 116.9 | -1.4 | 5.90E-08 | hypothetical protein |
| A1S_3476 | 20.6 | 55.9 | -1.4 | 1.80E-04 | secretory lipase |
| A1S_0057 | 79.9 | 190.8 | -1.3 | 9.50E-11 | capsular polysaccharide synthesis enzyme |
| A1S_0342 | 27 | 65.7 | -1.3 | 2.00E-04 | monovalent cation/H+ antiporter subunit C |
| A1S_0911 | 99.8 | 249.5 | -1.3 | 7.40E-15 | hypothetical protein |
| A1S_1228 | 1942.4 | 4707.3 | -1.3 | 3.50E-254 | cold shock protein |
| A1S_1384 | 19.1 | 47.2 | -1.3 | 1.60E-03 | CinA-like protein |
| A1S_1844 | 70.9 | 169.5 | -1.3 | 1.20E-09 | CatC3 |
| A1S_2125 | 56.9 | 141 | -1.3 | 1.10E-08 | VIC family potassium channel protein |
| A1S_2872 | 39.7 | 96.2 | -1.3 | 5.10E-06 | hypothetical protein |
| A1S_3294 | 59.6 | 147.7 | -1.3 | 4.70E-09 | TetR/AcrR family transcriptional regulator |
| A1S_1281 | 185.3 | 412.7 | -1.2 | 1.50E-19 | hypothetical protein |
| A1S_1296 | 193 | 450.5 | -1.2 | 3.20E-23 | hypothetical protein |
| A1S_1841 | 32.6 | 73 | -1.2 | 2.90E-04 | hypothetical protein |
| A1S_1843 | 176.8 | 408.1 | -1.2 | 1.10E-20 | muconate cycloisomerase I |
| A1S_2089 | 73.1 | 165.4 | -1.2 | 1.30E-08 | fimbrial usher protein |
| A1S_3410 | 35.5 | 81.6 | -1.2 | 7.40E-05 | acyltransferase |
| A1S_0534 | 492.1 | 1074.3 | -1.1 | 1.00E-47 | NADH-dependent enoyl-ACP reductase |
| A1S_0817 | 1011.4 | 2225.7 | -1.1 | 4.90E-100 | hypothetical protein |
| A1S_0913 | 163.9 | 347.6 | -1.1 | 4.50E-15 | hypothetical protein |
| A1S_1009 | 407.9 | 878.7 | -1.1 | 4.20E-38 | lipoprotein |
| A1S_1209 | 31 | 66.5 | -1.1 | 1.10E-03 | benzoate transport porin (BenP) |
| A1S_1210 | 86.3 | 190.5 | -1.1 | 2.30E-09 | major facilitator superfamily transporter |
| A1S_1230 | 50.3 | 105.3 | -1.1 | 4.30E-05 | phage putative head morphogenesis protein |
| A1S_1386 | 3269.4 | 6948.7 | -1.1 | 3.70E-290 | catalase |
| A1S_2088 | 40.8 | 84.5 | -1.1 | 3.40E-04 | hypothetical protein |
| A1S_2397 | 28.1 | 61.7 | -1.1 | 1.20E-03 | hypothetical protein |
| A1S_2559 | 150.4 | 322 | -1.1 | 2.70E-14 | hypothetical protein |
| A1S_2560 | 48.7 | 106.7 | -1.1 | 1.40E-05 | hypothetical protein |
| A1S_0297 | 359.7 | 723 | -1 | 5.20E-27 | hypothetical protein |
| A1S_0340 | 29.1 | 59.5 | -1 | 3.70E-03 | pH adaptation potassium efflux system E transmembrane protein |
| A1S_0803 | 619.4 | 1276.2 | -1 | 6.30E-50 | trehalose-6-phosphate synthase |
| A1S_1062 | 63.5 | 129.1 | -1 | 1.00E-05 | FMN oxidoreductase |
| A1S_1139 | 130.8 | 264.4 | -1 | 1.40E-10 | signal peptide |
| A1S_1199 | 424.6 | 875 | -1 | 1.70E-34 | glutathionine S-transferase |
| A1S_1258 | 226.6 | 455.3 | -1 | 2.70E-17 | hypothetical protein |
| A1S_1761 | 38.1 | 76.3 | -1 | 1.20E-03 | acetyltransferase |
| A1S_1850 | 33.9 | 69.6 | -1 | 1.50E-03 | esterase |
| A1S_1863 | 83.9 | 169.7 | -1 | 4.00E-07 | hypothetical protein |
| A1S_3225 | 117.5 | 235.8 | -1 | 2.30E-09 | sulfate permease |
| A1S_3297 | 1014.9 | 2070.6 | -1 | 6.30E-79 | outer membrane protein |

*^a^q value, corrected p value; the smaller q value means the more significant difference in gene.*

Table S3 A list of differentially expressed genes that are up-regulated in mutant Δ2811::FRT (log2.Fold_change>1)

| Gene_ID | Readcount  _mu | Readcount  _wt | log2.Fold  _change | qvalue^a^ | Gene_description |
| --- | --- | --- | --- | --- | --- |
| A1S_1792 | 99.5 | 6.2 | 4 | 1.70E-21 | nucleoside-diphosphate-sugar epimerase |
| A1S_1791 | 106.7 | 10.4 | 3.4 | 2.30E-20 | tartrate symporter MFS superfamily protein |
| A1S_1805 | 104 | 10.4 | 3.3 | 1.00E-19 | major facilitator superfamily transporter |
| A1S_1794 | 69.4 | 7.6 | 3.2 | 5.80E-13 | hypothetical protein |
| A1S_1790 | 54.3 | 7.3 | 2.9 | 1.50E-09 | 6-phosphogluconate dehydrogenase |
| A1S_1422 | 66.7 | 12.7 | 2.4 | 1.60E-09 | triphosphoribosyl-dephospho-CoA synthase |
| A1S_1806 | 79.9 | 15.1 | 2.4 | 2.60E-11 | senescence marker protein-30 |
| A1S_1426 | 62.5 | 14.9 | 2.1 | 1.10E-07 | phosphoribosyl-dephospho-CoA transferase |
| A1S_0671 | 582.6 | 143.7 | 2 | 1.20E-62 | protein tyrosine phosphatase |
| A1S_1505 | 256.2 | 65.6 | 2 | 5.40E-27 | hypothetical protein |
| A1S_1795 | 86.6 | 21.5 | 2 | 6.00E-10 | dihydroxy-acid dehydratase |
| A1S_2169 | 987.4 | 243.7 | 2 | 1.00E-105 | cytochrome o ubiquinol oxidase subunit IV |
| A1S_0169 | 1137.7 | 301.4 | 1.9 | 8.70E-114 | hypothetical protein |
| A1S_1186 | 3499.7 | 921.5 | 1.9 | 0.00E+00 | ATP-dependent protease Hsp 100 |
| A1S_1423 | 24.4 | 6.7 | 1.9 | 3.30E-03 | malonate decarboxylase subunit delta |
| A1S_0040 | 246.2 | 70.7 | 1.8 | 3.70E-23 | oxidoreductase |
| A1S_0041 | 173.1 | 50.8 | 1.8 | 3.70E-16 | linoleoyl-CoA desaturase |
| A1S_1926 | 539.7 | 156.6 | 1.8 | 2.50E-49 | hypothetical protein |
| A1S_1421 | 219.7 | 67.2 | 1.7 | 2.20E-19 | malonate decarboxylase subunit alpha |
| A1S_0401 | 83.4 | 26.8 | 1.6 | 1.60E-07 |  |
| A1S_0669 | 348.6 | 112 | 1.6 | 1.40E-28 | bile acid:sodium symporter |
| A1S_1424 | 70.9 | 23.1 | 1.6 | 1.90E-06 | malonate decarboxylase subunit beta |
| A1S_1425 | 70.4 | 22.9 | 1.6 | 2.10E-06 | malonate decarboxylase subunit gamma |
| A1S_1526 | 2354.6 | 765.6 | 1.6 | 7.40E-187 | hypothetical protein |
| A1S_2325 | 110.1 | 35.3 | 1.6 | 1.20E-09 | outer membrane protein |
| A1S_0095 | 1392.6 | 476 | 1.5 | 1.10E-103 | D-amino acid dehydrogenase small subunit |
| A1S_0661 | 465.4 | 161 | 1.5 | 1.70E-34 | phage integrase family protein |
| A1S_0816 | 616.5 | 216 | 1.5 | 1.10E-44 | 50S ribosomal protein L32 |
| A1S_1046 | 92.6 | 32.1 | 1.5 | 1.40E-07 | lysine exporter protein LysE/YggA |
| A1S_1390 | 167.8 | 59.9 | 1.5 | 1.80E-12 | hypothetical protein |
| A1S_2093 | 610.7 | 212.7 | 1.5 | 1.30E-44 | hypothetical protein |
| A1S_2839 | 95.3 | 34.6 | 1.5 | 2.60E-07 | hypothetical protein |
| A1S_3122 | 912.7 | 312.1 | 1.5 | 4.60E-68 | hypothetical protein |
| A1S_0078 | 127.1 | 48.1 | 1.4 | 6.40E-09 | hypothetical protein |
| A1S_0244 | 86.6 | 31.7 | 1.4 | 1.10E-06 | hypothetical protein |
| A1S_0496 | 198.8 | 76.1 | 1.4 | 3.20E-13 | phosphatidylglycerophosphatase B |
| A1S_0644 | 138.7 | 53.4 | 1.4 | 2.00E-09 | hypothetical protein |
| A1S_0672 | 112.5 | 42.6 | 1.4 | 5.30E-08 | resolvase |
| A1S_1030 | 4064.8 | 1519.9 | 1.4 | 8.10E-266 | DNA-binding ATP-dependent protease La |
| A1S_1427 | 88.1 | 33.5 | 1.4 | 1.90E-06 | malonate decarboxylase subunit epsilon |
| A1S_1429 | 83.4 | 32.1 | 1.4 | 4.60E-06 | malonate transporter |
| A1S_0663 | 79.1 | 32.4 | 1.3 | 2.50E-05 | DNA helicase |
| A1S_2599 | 200.9 | 79.4 | 1.3 | 9.70E-13 | hypothetical protein |
| A1S_0125 | 218.9 | 94 | 1.2 | 4.50E-12 | hypothetical protein |
| A1S_0170 | 892.3 | 388.3 | 1.2 | 1.60E-45 | outer membrane copper receptor (OprC) |
| A1S_0445 | 986.3 | 438.5 | 1.2 | 3.20E-48 | hypothetical protein |
| A1S_0650 | 115.1 | 49 | 1.2 | 6.80E-07 | conjugal transfer protein |
| A1S_0738 | 145.9 | 62.1 | 1.2 | 1.80E-08 | flavoprotein oxidoreductase |
| A1S_0893 | 184.5 | 78.9 | 1.2 | 2.20E-10 | hypothetical protein |
| A1S_1220 | 131.6 | 58.3 | 1.2 | 3.10E-07 | threonine efflux protein |
| A1S_1428 | 43.7 | 18.9 | 1.2 | 4.20E-03 | malonate transporter |
| A1S_1528 | 1559.7 | 657.8 | 1.2 | 8.70E-84 | bifunctional proline dehydrogenase/pyrroline-5-carboxylate dehydrogenase |
| A1S_2159 | 320 | 142.2 | 1.2 | 3.50E-16 | hypothetical protein |
| A1S_2416 | 204.6 | 89.8 | 1.2 | 6.50E-11 | hypothetical protein |
| A1S_2730 | 150.6 | 64.3 | 1.2 | 1.10E-08 | 50S ribosomal protein L27 |
| A1S_3148 | 59 | 26.4 | 1.2 | 1.10E-03 | NADPH specific quinone oxidoreductase |
| A1S_3160 | 62.2 | 27.7 | 1.2 | 7.00E-04 | lipase |
| A1S_3171 | 1943.7 | 847.2 | 1.2 | 2.90E-98 | DNA-directed RNA polymerase subunit omega |
| A1S_3224 | 631.3 | 282.6 | 1.2 | 1.00E-30 | acyl coenzyme A reductase |
| A1S_0220 | 306.5 | 147.5 | 1.1 | 2.50E-13 | transcriptional regulator |
| A1S_0360 | 8314.7 | 3952.5 | 1.1 | 0.00E+00 | 30S ribosomal protein S15 |
| A1S_0630 | 211 | 99.2 | 1.1 | 7.10E-10 | hypothetical protein |
| A1S_0633 | 549.3 | 262.1 | 1.1 | 1.40E-23 | hypothetical protein |
| A1S_0640 | 134.7 | 63.2 | 1.1 | 1.10E-06 | hypothetical protein |
| A1S_0645 | 186.6 | 87.4 | 1.1 | 6.40E-09 | hypothetical protein |
| A1S_0675 | 982.6 | 463.5 | 1.1 | 1.10E-42 | dihydropteroate synthase |
| A1S_1724 | 121.5 | 55.9 | 1.1 | 2.40E-06 | major facilitator superfamily alpha-ketoglutarate permease |
| A1S_1932 | 2704.5 | 1275.3 | 1.1 | 1.20E-116 | hypothetical protein |
| A1S_1933 | 2823.6 | 1300.1 | 1.1 | 5.40E-128 | hypothetical protein |
| A1S_2291 | 105.4 | 50.6 | 1.1 | 3.30E-05 | hypothetical protein |
| A1S_2458 | 767.4 | 348.6 | 1.1 | 3.70E-36 | fatty acid desaturase |
| A1S_2459 | 1000.3 | 451.1 | 1.1 | 1.40E-47 | oxidoreductase |
| A1S_2598 | 1549.3 | 717.1 | 1.1 | 1.20E-69 | RNA polymerase factor sigma-70 |
| A1S_3440 | 140.3 | 64.6 | 1.1 | 3.80E-07 | MFS family transporter |
| A1S_0011 | 537.9 | 262.4 | 1 | 4.90E-22 | hypothetical protein |
| A1S_0490 | 670.2 | 333.3 | 1 | 3.80E-26 | hydrolase |
| A1S_0642 | 201.4 | 99.2 | 1 | 1.20E-08 | hypothetical protein |
| A1S_0690 | 1786 | 876.7 | 1 | 1.20E-70 | protein FilA |
| A1S_0879 | 149.6 | 72.7 | 1 | 7.50E-07 | cell division topological specificity factor |
| A1S_2296 | 220.2 | 107.4 | 1 | 1.50E-09 | protease |

*^a^q value, corrected p value; the smaller q value means the more significant difference in gene.*

Table S4 RT-PCR results of other tested genes

|  | Δ2811/17978 ratio(n=3) |
| --- | --- |
| A1S_0232(*pilR*) | 0.6615 |
| A1S_0234(*pilR*) | 0.6965 |
| A1S_0235(*pilS*) | 0.6364 |
| A1S_0327(*pilD*) | 0.6212 |
| A1S_0328(*pilC*) | 0.6721 |
| A1S_0329(*pilB*) | 0.7228 |
| A1S_0500(*pilF*) | 0.7875 |
| A1S_0896(*pilU*) | 0.7152 |
| A1S_0897(*pilT*) | 0.724 |
| A1S_1559(*pilZ*) | 0.8537 |
| A1S_2812(*pilJ*) | 0.8814 |
| A1S_2813(*pilI*) | 0.6597 |
| A1S_2814(*pilH*) | 0.7031 |
| A1S_2815(*pilG*) | 0.7918 |
| A1S_3165(*pilE*) | 0.8972 |
| A1S_3166(*pilE*) | 0.9384 |
| A1S_3167(*pilY1*) | 0.9935 |
| A1S_3168(*pilW*) | 1.082 |
| A1S_3177(*pilA*) | 0.9103 |
| A1S_3191(*pilQ*) | 0.9715 |
| A1S_3192(*pilP*) | 1.051 |
| A1S_3193(*pilO*) | 1.194 |
| A1S_3194(*pilN*) | 1.227 |
| A1S_3195(*pilM*) | 1.138 |

The ratio >2 or <0.5 is considered changed significantly.
